# Selective Modulation of α5 GABA_A_ Receptors Exacerbates Aberrant Inhibition at Key Hippocampal Neuronal Circuits in *APP* Mouse Model of Alzheimer’s Disease

**DOI:** 10.3389/fncel.2020.568194

**Published:** 2020-11-11

**Authors:** Alexandra L. Petrache, Archie A. Khan, Martin W. Nicholson, Alessandra Monaco, Martyna Kuta-Siejkowska, Shozeb Haider, Stephen Hilton, Jasmina N. Jovanovic, Afia B. Ali

**Affiliations:** UCL School of Pharmacy, London, United Kingdom

**Keywords:** Alzheimer’s disease, GABA_A_ receptors, synaptic, interneurons, hippocampus

## Abstract

Selective negative allosteric modulators (NAMs), targeting α5 subunit-containing GABA_A_ receptors (GABA_A_Rs) as potential therapeutic targets for disorders associated with cognitive deficits, including Alzheimer’s disease (AD), continually fail clinical trials. We investigated whether this was due to the change in the expression of α5 GABA_A_Rs, consequently altering synaptic function during AD pathogenesis. Using medicinal chemistry and computational modeling, we developed aqueous soluble hybrids of 6,6-dimethyl-3-(2-hydroxyethyl) thio-1-(thiazol-2-yl)-6,7-dihydro-2-benzothiophene-4(5H)-one, that demonstrated selective binding and high negative allosteric modulation, specifically for the α5 GABA_A_R subtypes in constructed HEK293 stable cell-lines. Using a knock-in mouse model of AD (*APP*^NL−F/NL−F^), which expresses a mutant form of human amyloid-β (Aβ), we performed immunofluorescence studies combined with electrophysiological whole-cell recordings to investigate the effects of our key molecule, α5-SOP002 in the hippocampal CA1 region. In aged *APP*^NL−F/NL−F^ mice, selective preservation of α5 GABA_A_Rs was observed in, calretinin- (CR), cholecystokinin- (CCK), somatostatin- (SST) expressing interneurons, and pyramidal cells. Previously, we reported that CR dis-inhibitory interneurons, specialized in regulating other interneurons displayed abnormally high levels of synaptic inhibition in the *APP*^NL−F/NL−F^ mouse model, here we show that this excessive inhibition was “normalized” to control values with bath-applied α5-SOP002 (1 μM). However, α5-SOP002, further *impaired* inhibition onto CCK and pyramidal cells that were already largely compromised by exhibiting a *deficit* of inhibition in the AD model. In summary, using a multi-disciplinary approach, we show that exposure to α5 GABA_A_R NAMs may further compromise aberrant synapses in AD. We, therefore, suggest that the α5 GABA_A_R is not a suitable therapeutic target for the treatment of AD or other cognitive deficits due to the widespread neuronal-networks that use α5 GABA_A_Rs.

## Introduction

Over the last few decades, considerable focus has been on negative allosteric modulators (NAMs; previously referred to as inverse agonists) of the benzodiazepine site of *γ*-aminobutyric acid receptors (GABA_A_Rs) as a potential therapeutic target for cognitive impairment in temporal lobe epilepsy (TLE), Huntington’s disease, Down’s syndrome, schizophrenia and the most common form of dementia, Alzheimer’s disease (AD), which constitutes one of the most significant health problems confronting societies with an aging population.

The ionotropic GABA_A_R family are heteropentameric structures consisting of a combination of five subunits (Sieghart and Sperk, 2002) with the α–subunit being clinically relevant, as it controls the pharmacological profile of GABA_A_ Rs (McKernan and Whiting, 1996). Since the understanding that distinct pharmacological properties of the GABA_A_R are reliant on the fact that different brain regions and cell types contain various subunit compositions, NAMs of the GABA_A_R at the subunit level have been widely studied. In particular, GABA_A_Rs containing the α*5-subunit* have been of interest, given their role in learning and memory as evidenced by various studies (Collinson et al., 2002; Crestani et al., 2002; Caraiscos et al., 2004; Yee et al., 2004; Dawson et al., 2006; Ghafari et al., 2017).

The hippocampus plays a critical role in memory formation and retrieval and is significantly affected in AD, which is characterized by short-term memory deficits as one of the first symptoms of the disease (Price et al., 2001). The strong evidence to suggest hippocampal preferential distribution of the α5-containing GABA_A_R sub-type (Quirk et al., 1996), together with its diverse pathology in memory deficit-related disease, and particularly, its preservation in human brains of AD patients (Howell et al., 2000; Rissman et al., 2007), has led many researchers to test several α5 subunit-selective compounds for their potential cognition-enhancing effects (Liu et al., 1996; Quirk et al., 1996; Sternfeld et al., 2004; Savić et al., 2008).

Originally, Merck, Sharp, and Dohme (MSD) developed the first GABA_A_R NAM, known as α5IA, with high efficacy at the GABA_A_ α5 receptor sub-type without being an anxiogenic agent (Atack et al., 2006). Following the development of this compound by MSD, several other nootropic drugs (α5 sub-type selective NAMs) have been developed (e.g., RO4938581; Ballard et al., 2009). Many of these studies reported an impressive pharmacological profile of this compounds and their potential as cognitive enhancers without CNS-mediated adverse effects (Chambers et al., 2003; Collinson et al., 2006; Dawson et al., 2006; Ballard et al., 2009; Braudeau et al., 2011; Martinez-Cue et al., 2014; Duchon et al., 2019; Eimerbrink et al., 2019). These studies were initially implemented in rodent models, and unfortunately, these results were not reproducible in human subjects/patients to the same extent. Several key molecules consistently failed clinical trials at different phases including Basmisanil (code, RO5186582), a5IA (Atack, 2010), and MRK-016 (Atack et al., 2009). Basmisanil entered through Phase 1 and Phase 2 of clinical trials for Down’s syndrome but failed during Phase 2 due to a lack of efficacy in adults and adolescents. It appears that despite a5IA and MRK-016 demonstrating tolerance in young males, some of these molecules were poorly tolerated in elderly patients with no cognitive improvement (Atack, 2010), thus reducing the viability of α5 as a therapeutic target. Although these molecules were shown to be selective for α5 subunit-containing GABA_A_Rs, the lack of efficacy and poor tolerance in human patients could be related to poor brain penetration of the molecules or an age-related effect.

Whether this failure was due to low drug potency/bioavailability or due to a general lack of understanding of the synaptic mechanisms involving α5 receptors during the pathogenesis of the disease is currently unclear. To address these issues, we synthesized a novel water-soluble α5 GABA_A_R selective NAM. These receptor subtypes are located in hippocampal extrasynaptic sites, as well as synaptic sites of postsynaptic pyramidal cells (Serwanski et al., 2006; Ali and Thomson, 2008; Glykys et al., 2008). Although it has been shown that dendrite-targeting interneuron populations elicit α5 GABA_A_R-mediated inhibition in pyramidal cells (Ali and Thomson, 2008), it is unclear whether the α5 receptor subtype was expressed on inhibitory interneurons themselves. This was of particular interest, as we have shown previously, using the *APP*^NL−F/NL−F^ mouse, the first β-amyloid precursor protein (*APP*) knock-in mouse AD model that is thought to be able to recapitulate the human condition more accurately (see Sasaguri et al., 2017), that synaptic excitability is disrupted in various cortical regions, including the CA1 region (Petrache et al., 2019), and that this could be related to the alteration of three key modulatory interneuron populations namely; calretinin- (CR), cholecystokinin- (CCK), and somatostatin- (SST) expressing interneurons (Shi et al., 2019). We investigated whether these key modulatory interneurons located in CA1 stratum oriens (SO), stratum radiatum (SR), together with principal pyramidal cells in stratum pyramidale (SP), expressed the α5 subunit-containing GABA_A_Rs, in the *APP*^NL−F/NL−F^ model, age-matched to wild-type control mice, and then characterized the synaptic effects of our newly-developed α5 compound in these four subtypes of neurons.

## Materials and Methods

### Development of α5-SOP002

**Figure 1:**
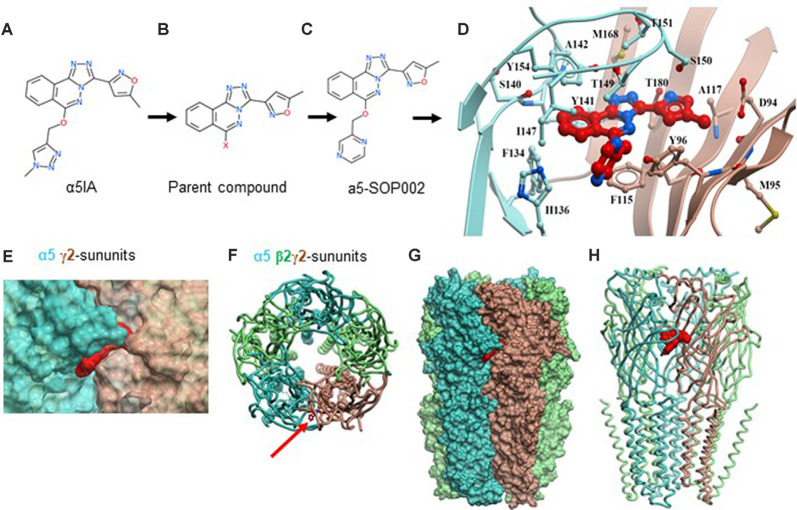
Developing negative allosteric modulator (NAM), α5-SOP002. **(A–C)** Optimization of α5IA to α5-SOP002. **(D)** Detailed interactions of α5-SOP002 at the GABA_A_R binding site located at the interface between subunit α5 (blue) and γ2 (brown). **(E)** Surface representation of SH-AI-SOP002 (red) interacting with the α5 GABA_A_R at the α5 (blue) and γ2 (brown) subunits’ interface. **(F)** The upper view of the α5 GABA_A_ subtype is represented by ribbons. The red arrow points at α5-SOP002. Subunits α5 are shown in blue, β3 in green, and γ2 in brown. **(G)** Surface and **(H)** ribbon representation of the α5 GABA_A_R.

We re-synthesized 6,6-dimethyl-3-(2-hydroxyethyl) thio-1-(thiazol-2-yl)-6,7-dihydro-2-benzothiophene-4(5H)-one that has demonstrated selectivity for the benzodiazepine binding site and high negative allosteric modulation for the α5 GABA_A_R sub-type following its published route, from the parent compound (Sternfeld et al., 2004; Atack, 2010) to develop hybrid derivatives (parent compound, shown in Figure 1A), full details of the synthetic steps are detailed in Supplementary Scheme 1 (see also Sung and Lee, 1992). There were two main sites for modification, which we explored *via* replacement of the triazole moiety or the oxazole which enabled us to explore late-stage modification to synthesize hybrid analogs to improve potency as a NAM acting on α5 GABA_A_Rs.

### Computational Modeling

The structure of the α5 subunits contained in the A-type γ-aminobutyric acid receptor (GABA_A_R) subtype formed by two α5, two β3, and one γ2 subunits was modeled based on the Cryo-EM structure 6A96 downloaded from the protein data bank^1^. Then, the complete GABA_A_R was modeled. Potential pockets that were large enough to bind the ligands were identified using the icmPocketfinder tool present in the ICM-Pro software^2^. The pocket selected was present at the interface of the subunits α5 and γ2 and was analogous to that which binds benzodiazepine in the GABA_A_R, the human β3 homopentamer (PDB id: 4COF). The volume of the pocket was 435.6 Å^3^.

The ligands were sketched using the LigEdit module and docked in the receptor using the docking module. The template-based docking protocol was used. The spatial orientation of benzodiazepine was selected as a reference template to dock the compounds. Grid maps were generated around the template, which defined a binding site encompassed in a grid of 20 × 20 × 20 Å^3^. Docking was run with an effort of 5, storing all alternative conformations. A maximum of 25 docked conformations was generated. The final confirmation was chosen based on the strongest interaction energy. Visualization of the docked poses was done by using the ICM-Pro Molsoft molecular modeling package.

### Preparation of Stable HEK293 Cell Lines Expressing GABA_A_Rs

To test the target selectivity of α5-SOP002, a stable cell line of HEK293 cells expressing α5β2γ2 subunits of the GABA_A_R was developed using the previously established method based on antibiotic selection (Brown et al., 2016). HEK293 cells (2 × 106) were transfected using Lipofectamine LTX (catalog no. 15338–100, Invitrogen) with the α5 pcDNA3.1(+) construct, incorporating the G418 disulfate (Neomycin) resistance gene and β2 pcDNA3.1(+) construct, incorporating the Zeocin resistance gene. Cells were subsequently plated at the ratios of 1:3, 1:5, 1:7, 1:10, 1:15, and 1:20, and selected with G418 (Neomycin; catalog no. G5013, Sigma–Aldrich) and Zeocin (catalog no. R25001 Gibco) antibiotics (both at 800 μg/ml) until colonies were formed. After 7 days, ~5–20 single colonies were selected and gradually scaled up. The clone expressing the highest level of GABA_A_R α5 and β2 subunits, as well as the previously established α2β2-HEK293 (Brown et al., 2016) stable cell line were further transfected with the γ2 pcDNA3.1(+) construct, incorporating the Hygromycin resistance gene, to produce triple cell lines. The expression of all three subunits was characterized by immunoblotting and immunocytochemistry. The α1β2γ2-HEK293 was characterized previously (Fuchs et al., 2013).

### Experimental Animals

All of the procedures in this study were carried out following the British Home Office regulations under the Animal Scientific Procedure Act 1986, under the project license PPL: P1ADA633A held by the principal investigator, Dr. Afia Ali. All procedures were approved by both internal and external UCL ethics committees and following the ARRIVE guidelines for reporting experiments involving animals (McGrath et al., 2010). A total of ~100 male animals (disease model and wild-type) were used in this study. The animals had *ad-libitum* access to food and water and were reared in cages of a maximum of five inhabitants, with a day: night cycle of 12: 12 h.

The knock-in *APP*^NL−F/NL−F^ AD mouse model was used for experiments (Saito et al., 2014), which consists of the introduction of two familial AD (FAD) mutations: KM670/671NL and I716F. The former, identified as the Swedish mutation, increases β-site cleavage of APP to produce elevated amounts of both Aβ_40_ and Aβ_42_, whereas the latter, known as the Beyreuther/Iberian mutation, promotes γ-site cleavage at C-terminal position 42, thereby increasing the Aβ_42_/Aβ_40_ ratio in favor of the more hydrophobic Aβ_42_ (Saito et al., 2014). Both features are key to the integrity of the disease phenotype. The knock-in line was crossed with C57BL/6 mice, and male *APP*^NL−F/NL−F^ and age-matched wild-type (C57BL/6) mice from the same breeding were used as control at 10–18 months (age ranges of mice for neuroanatomy and electrophysiology experiments were; 12–18 months and 10–12 months, respectively).

Animals were genotyped *via* standard polymerase chain reaction using the following four primers: 5′-ATCTCGGAAGTGAAGATG-3′, 5′-TGTAGATGAGAACTTAAC-3′, 5′-ATCTCGGAAGTGAATCTA-3′, and 5′-CGTATAATGTATGCTATACGAAG-3′ as previously described (Saito et al., 2014). Further details of the rationale for selecting this mouse model can be found in Petrache et al. (2019).

For the *in vivo* radial arm maze (RAM) memory test, male Wistar rats (Harlan, UK) at post-natal days 20–27 with the same housing conditions as the mice were used. The rats were weighed, handled, and monitored daily systematically during the memory test.

### Tissue Collection and Preparation

Male rodents were anesthetized by an intraperitoneal injection of 60 mg/kg phenobarbital and perfused transcardially with artificial cerebrospinal fluid (ACSF) containing sucrose. The level of anesthesia was monitored using pedal and tail pinch reflexes, rate, depth, and pattern of respiration through observation and color of mucous membranes and skin. The ACSF comprised of (in mM): 248 sucrose, 3.3 KCl, 1.4 NaH_2_PO_4_, 2.5 CaCl_2_, 1.2 MgCl_2_, 25.5 NaHCO_3_, and 15 glucose, which was bubbled with 95% O_2_ and 5% CO_2_. The animals were then decapitated and the brain removed and coronal sections hippocampus containing the neocortex ~300 μm thick—were cut in ice-cold standard ACSF using an automated vibratome (Leica, Germany). This standard ACSF contained (in mM): 121 NaCl, 2.5 KCl, 1.3 NaH_2_PO_4_, 2 CaCl_2_, 1 MgCl_2_, 20 glucose and 26 NaHCO_3_, equilibrated with 95% O_2_ and 5% CO_2_. Slices were incubated in ACSF for 1 h at room temperature (20–23°C) before recording. Brain slices were placed in a submerged chamber and superfused with ACSF at a rate of 1–2 ml min^1^ for electrophysiological recordings. For neuroanatomical studies, brains were immediately fixed after perfusion in 4% paraformaldehyde (PFA) plus 0.2% picric acid in 0.1 M phosphate buffer (PB) for 24 h before sectioning.

### *In vitro* Brain Slice Electrophysiology

All whole-cell recordings were performed using patch electrodes made from filamented borosilicate glass capillaries (Harvard Apparatus, UK) using a laser puller (Sutter Instruments, Novato, CA, USA), with resistances of 8–11 MΩ, and were visually aided by IR-DIC microscopy (Optizoom, Nikon, USA).

#### Whole-Cell Patch-Clamp Recordings of HEK293 Cells

Electrophysiological recordings of HEK293 cells stably expressing GABA_A_Rs were performed in a whole-cell, voltage-clamp mode. The chamber containing coverslips with the cell line was continuously superfused at a flow rate of 1.8 ml/min with the extracellular medium composed of 130 mM NaCl, 4 mM KCl, 10 mM HEPES, 20 mM NaHCO_3_, 10 mM glucose, 1 mM MgCl_2_, and 2 mM CaCl_2_, and was equilibrated with 5% CO_2_/95% O_2_ and maintained at room temperature (~21–25°C). The electrodes were filled with an intracellular solution containing (in mM), 130 KCl, 3 NaCl, 4.5 phosphocreatine, 10 HEPES, 1 EGTA, 3.5 Na-ATP, 0.45 Na-GTP, and 2 MgCl_2_ (adjusted to pH 7.2 with KOH, 290–300 mOsmol/l), and had a final resistance of 3–8 MΩ. To test the target selectivity of α5-SOP002, the responsiveness to applied GABA was investigated and measured in HEK293 cells stably expressing either, α5β2γ2, α1β2γ2 or α2β2γ2 subunits of GABA_A_Rs. The pharmacological properties of the expressed receptors were investigated by puffer-application of GABA (1 μM; Tocris Bioscience, UK) and subsequent bath-application of α5-SOP002 (0.5–1 μM), followed by diazepam (1 μM, Tocris Bioscience, UK). The change in voltage after the GABA puff application response was recorded. The statistical test used was one-way ANOVA with a 95% confidence interval.

#### Whole-Cell Patch-Clamp of Neurons in Acute Hippocampal Brain Slices

Whole-cell somatic recordings were performed using patch electrodes filled with a solution containing (in mM): 134 K gluconate, 10 HEPES, 10 phosphocreatine, 2 Na_2_ATP, 0.2 Na_2_GTP, and 0.2% w/v biocytin.

CA1 pyramidal cells and interneurons in SR and stratum lacunosum moleculare (SLM) were selected for recording based on the shape of their soma using video microscopy under near-infrared differential interference contrast illumination. Cells were further characterized by their electrophysiological properties obtained from injecting a series of 500 ms depolarizing and hyperpolarizing current pulses and identified post-recording anatomically, as described previously in detail (Khan et al., 2018).

Spontaneous postsynaptic potentials were recorded from passive membrane responses and mixed spontaneous excitatory postsynaptic potentials (sEPSPs) and spontaneous inhibitory postsynaptic potentials (sIPSPs) were collected in 60-s frame samples, repeated at 0.33 Hz. Recordings were carried out under the current-clamp mode of operation (NPI SEC 05LX amplifier; NPI electronics, Germany), low pass filtered at 2 kHz and digitized at 5 kHz using a CED 1401 interface (Cambridge Electronic Design, UK). Input resistance was monitored throughout experiments using a hyperpolarizing current step (−10 pA, 10 ms). Signal (Cambridge Electronic Design, UK) was used to acquire recordings and generate current steps. The average amplitudes of spontaneous events and their frequency were measured manually from single sweep data sets of 60-s recordings, including a total sweep range of 30–50 frames (i.e., 30–50 min of recording); values below the baseline level of 0.1 mV were considered as noise, see Ali and Nelson (2006).

Paired whole-cell somatic recordings were obtained between CA1 CR interneurons in SR (for inhibitory connections). Unitary inhibitory postsynaptic potentials (IPSPs) were elicited by a depolarizing current step into the presynaptic neuron (+0.05 nA, 5–10 ms) repeated at 0.33 Hz. The peak IPSP amplitudes and width at half-amplitude measurements were obtained from averages including 100–200 unitary synaptic events.

Drugs for *in vitro* pharmacological studies on brain slices, zolpidem (Sigma–Aldrich, UK, 0.4 μM, dissolved first in ethanol to a final bath ethanol dilution of 1:20,000); α5-SOP002 (1–1.5 μM); diazepam (RBI, Poole UK; 1–2 μM, dissolved in ethanol to a final bath ethanol dilution of 1:5,000) were bath-applied. The α5-SOP002 concentration used was similar to the previously published parent compound, α5IA (1–1.5 μM); this was within the range of *in vitro* efficacy at which it is reported to act as an inverse agonist (NAM) with efficacy selective for α5 containing GABA_A_Rs (Collinson et al., 2006; Dawson et al., 2006). The concentration of zolpidem used produces near-maximal effects on α1-containing receptors but submaximal effects on α2/3-containing receptors (*K*_d_ 0.2 μM for α1–containing receptors; 1.5 μM for α3 containing receptors; Munakata et al., 1998).

### Neuroanatomical Procedures and Analysis

#### Recovery of Biocytin Labeled-Cells Post Electrophysiological Recordings

After electrophysiological recordings with pharmacological protocols, the slices were only suitable for biocytin recovery due to the long recording in the range of 45–90 min. Slices were fixed in 4% PFA plus 0.2% picric acid in 0.1 M PB for 24 h and then re-sectioned at 70 μm. Slices were then incubated in ABC overnight at 4°C, followed by the above DAB protocol. Cells were identified using a Leica DMR microscope.

#### Immunofluorescence Procedures, Confocal Image Acquisition, and Analysis of CA1 Neurons

Slices obtained from approximately the same medial level in CA1 were incubated as described previously (Petrache et al., 2019), using GABA_A_R α5 primary antibody (Abcam, Cambridge, MA, USA, raised in mouse, 1:100) incubated concomitantly with the primary antibody targeting one of the following: calretinin (Swant, raised in goat, 1:1,000), somatostatin (Santa Cruz Biotechnology, Santa Cruz, CA, USA, raised in rabbit, 1:500), cholecystokinin (Frontier Institute, raised in rabbit, 1:1,000) or CaMKII-α (Invitrogen, raised in goat, 1:100). The secondary antibodies used were as follows: FITC (Sigma–Aldrich, anti-mouse, 1:200), Texas Red (Invitrogen, anti-rabbit/anti-mouse, 1:500) or Alexa Fluor 488 (Abcam, Cambridge, MA, USA, anti-goat, 1:500). The sections were counterstained with the nuclear stain, DAPI (Sigma–Aldrich, 1:1,000).

Images were acquired at 63× magnification using a ZEISS LSM 880 confocal microscope and processed using Zen Black 2009. When imaging, we maintained a consistent pinhole, exposure time, and light intensity settings between experiments. Collapsed *Z*-stacks were imported into Fiji (ImageJ) as .tif files and split into individual channels. If needed, the background was removed using the *Background subtraction* function in ImageJ, and this was applied to all channels for a given data set. In the channel corresponding to the cell staining, the outline of the cells of interest was drawn manually to obtain regions of interest (ROIs). The *Coloc2* plugin was then used to obtain Pearson’s R coefficient as a measure of colocalization between the channels corresponding to the ROIs and the α5 subunit, and Fisher’s transformation was applied to convert the coefficients to a normal distribution. The results so obtained were then averaged separately for wild-type and *APP*^NL−F/NL−F^ animals, respectively, for each of the cells of interest. There were no age-dependent differences observed for either wild-type and *APP*^NL−F/NL−F^ animals during confocal analysis, however, the data presented for the expression of α-5 GABA_A_Rs were obtained from individual animals in the age bracket of 12–18 months (*n* = 7).

### *In vivo* RAM Memory Test

A RAM was used to test the *in vivo* effects of a5-SOP002 on memory. The RAM consisted of eight identical arms and a circular platform. The maze was placed on a Table 50 cm above the floor with a digital camera recorder mounted to the ceiling directly above. All rats were first habituated in the maze for 5 days with up to two sessions of 10–30 min per day with either food scattered throughout the maze, food scattered only in the arms, and food scattered in three designated arms. The rats were further trained for another 5 days by assigning a hippocampal-dependent memory task. Three out of eight arms of the maze were baited with food. The three designated arms in which the food bait was placed were randomized between each rat. The rats were placed at the center of the platform of the maze and allowed to retrieve food reward from the baited arms. Completion of the training was accepted if one of these criteria were met: (i) the training lasted more than 10 min; or (ii) all eight arms of the maze were visited. The fourth day of the training was assigned as the information phase where we assumed the rats have learned the task. The final day of the training was assigned as the test phase. The rats were administered a drug treatment (a5-SOP002 or L-655, 708, 1 μM in 5% DMSO) or saline (sodium chloride BP 0.9% w/v) 2 h before the beginning of the task at a dose of 1 mg/kg i.p. A 2-day interval was kept before the test day. The total time the rats took to complete the task was recorded and tracked using a video tracking software ANY-maze (Stoelting Co., Wood Dale, IL, USA).

### Statistical Analyses

All data values are given as the mean ± standard error of the mean (SEM) unless otherwise stated. Before statistical analysis, normality and outlier tests were conducted. For comparisons between multiple groups of data, one-way or two-way ANOVA with a 95% confidence interval was used followed by a *post hoc* Tukey’s or Bonferroni’s test for multiple comparisons.

Statistical analysis for the electrophysiology in the *APP*^NL−F/NL−F^ model and the immunofluorescence data was conducted using GraphPad Prism version 8.0.0 for Windows, GraphPad Software, San Diego, CA, USA, GraphPad.

All statistical analyses were conducted using the statistical package Origin Pro 2016 SR1. Statistical significance was accepted where *P* < 0.05 (*P < 0.05, **P < 0.01, ***P < 0.001, ****P < 0.0001). The *“n”* is given as the number of observations and the number of animals used unless otherwise stated.

## Results

In this study, we initially re-synthesized a water-soluble α5 GABA_A_R-selective compound NAM, α5-SOP002 and determined its selectivity using HEK293 cells lines stably expressing α5β2γ2-, α2β2γ2-, or α1β2γ2-GABA_A_Rs. To identify changes in the expression pattern of α5 GABA_A_R during a disease that is characterized by cognitive deficits, we used an AD mouse model and wild-type mice at 10–12 months, when the typical hallmarks of AD in the hippocampus are present, including synaptic loss, accumulation of amyloid-β (Aβ) and proliferation of reactive astrocytes and microglia (Saito et al., 2014; Petrache et al., 2019). The effects of α5-SOP002 on inhibitory synaptic potentials recorded in the identified cells that co-expressed α5 GABA_A_R were investigated.

### The Development of the α5-SOP002 Compound

We initially developed four hybrid analogs of this compound with an array of biological activity ranging from inactive controls to highly potent derivatives resulting in, α5-SOP002 (Figures 1A–C, see also Supplementary Scheme 1).

The structure of the α5 subunits contained in the α5 GABA_A_R was modeled and later used to generate the GABA_A_R subtype containing two α5, two β3, and one γ2 subunits. Once a reliable model was obtained, our key compound, α5-SOP002 was docked into the interface of subunit α5 (Figures 1D–H) and subunit γ2, obtaining the best binding mode with a VlsScore of −20.35.

Overall, α5-SOP002 indicated good aqueous solubility and good blood-brain barrier penetration as evidenced by the spatial memory recall experiments in rats following intraperitoneal injection (i.p.; Supplementary Scheme 1). The Supplementary Section, which compares *in vivo* spatial memory tests (Becker et al., 1980) and *in vitro* paired whole recording data from 25 to 28 day old rats using α5-SOP002 and the published analog L-655, 708 (a similar compound to α5IA originally developed by Merck Sharp and Dome (UK) and available from Tocris (UK) were described. *In vivo*, spatial memory recall experiments were not repeated in the mouse lines due to the conclusions reached from the results (see below).

### α5-SOP002 Selectively Targets α5 Subunits of GABA_A_Rs

**Figure 2:**
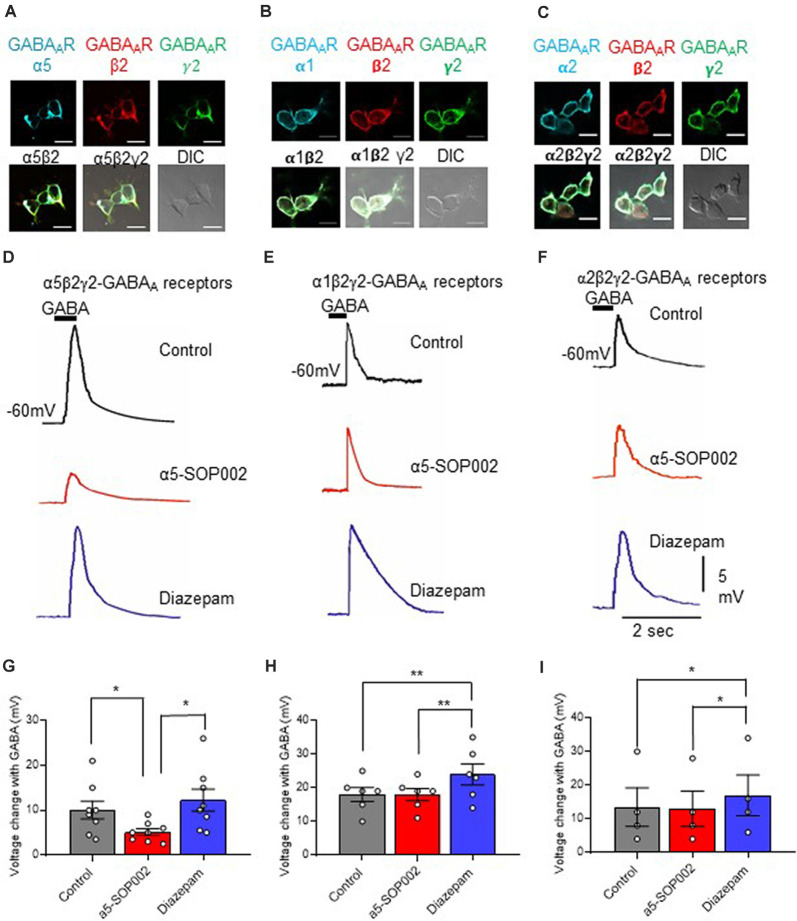
α5-SOP002 selectively targets α5 subunits of GABA_A_Rs. Whole-cell recordings in α5β2γ2-, α1β2γ2-, and α2β2γ2-HEK293 cells. HEK293 cells stably expressing α5β2γ2- **(A)**, α1β2γ2- **(B)**, or α2β2γ2-GABA_A_Rs **(C)**. Immunofluorescent imaging with a 40× oil immersion objective lens shows cell surface expression of α5, α1 or α2- (cyan), β2- (red), and γ2-GABA_A_R subunits (green). **(A–C)** also show all the three channels merged showing α-, β2-, and γ2-GABA_A_R subunit co-localization at the cell surface (white) along with the differential interference contrast microscopy (DIC) image of the cells. The scale bar represents 10 μm. All three stable cell lines responded to 10 μM puff-applied GABA **(D–F)** in control extracellular solution (black traces), an extracellular solution containing 1 μM α5-SOP002 (red traces), and subsequent bath application of diazepam (blue traces) at a holding membrane potential of −60 mV. The corresponding plots for α5β2γ2-HEK293 cells **(G–I)** show the changes in voltage changes in response to 10 μM GABA puffed locally, in the presence of bath-applied α5-SOP002, and, subsequent addition of diazepam. Only the α5β2γ2-HEK293 cells showed an inverse agonist effect (response to GABA) of α5-SOP002. All three cell lines, however, showed an enhancement of response to GABA in the presence of diazepam. Statistically significant data are shown with *P < 0.05 and **P < 0.01.

An α5β2γ2-HEK293 cell line was developed to investigate the selectively of α5-SOP002 towards the α5-containing GABA_A_Rs. The cell surface expression of all three GABA_A_R subunits in this cell line was characterized using immunocytochemistry (Figure 2A) with subunit-specific antibodies. The responsiveness of the α5β2γ2-HEK293 stable cell line to 10 μM puff-applied GABA in the presence α5-SOP002, confirmed its activity as a NAM (i.e., inverse agonist) of these receptors. Subsequent bath addition of diazepam, followed by puff-applied GABA resulted in an enhanced voltage change and demonstrated the presence of functional α5β2γ2-GABA_A_Rs at the cell surface (Figure 2D). These experiments were repeated using the α1β2γ2-HEK293 and α2β2γ2-HEK293 stable cell lines to test the specificity of α5-SOP002. The cell surface expression of α1β2γ2-and α2β2γ2-GABA_A_Rs was also demonstrated using immunocytochemistry with subunit-specific antibodies (Figures 2B,C), as shown previously (Fuchs et al., 2013; Brown et al., 2016).

HEK293 cells expressing α5β2γ2-GABA_A_Rs responded to GABA (10 μM), puff-applied (5 s) in proximity, with a large hyperpolarization, recorded at a membrane holding potential of −60 mV. This was also recorded in the α1β2γ2-HEK293 and α2β2-HEK293 stable cell lines (Figures 2E,F).

The response of the three cell lines to GABA was measured and the changes of the response after bath-application of 1 μM α5-SOP002, followed by puff-application of GABA after subsequent bath application (to extracellular solution) of the broad spectrum GABA_A_R modulator, diazepam (1 μM) was also analyzed (Figures 2D–F).

Bath-application of α5-SOP002 (1 μM) significantly reduced the hyperpolarizing GABA inhibitory response in cells expressing α5β2γ2- GABA_A_Rs (mean ± SEM: control GABA: 10.0 ± 5.0 mV; α5-SOP002: 5.12 ± 2.2 mV; *P* < 0.05, *n = 8*), while bath application of diazepam had an opposite effect leading to a significant enhancement of GABA response (12.26 ± 6.94, *P* < 0.05, *n =* 8, one-way ANOVA; Figure 2G). In contrast, there were no significant changes in the puff-applied GABA response in the presence of α5-SOP002 in cells expressing α1β2γ2-GABA_A_Rs (control GABA: 18.0 ± 5.0 mV; α5-SOP002: 18.0 ± 4.5, *n* = 6; Figure 2E) or α2β2γ2-HEK293 (control GABA: 13.5 ± 11.5 mV; α5-SOP002: 13.0 ± 10.5 mV, *n* = 6; Figure 2F). Puff-application GABA in the presence of diazepam enhanced the hyperpolarizing inhibitory GABA response in both, α1β2γ2-HEK293 (24.0 ± 7.6 mV, *P* < 0.01, *n =* 6) and α2β2γ2-HEK293 cells (17.0 ± 12.0, *P* < 0.05, *n =* 6; Figures 2H,I). This confirmed the selectivity of α5-SOP002 towards GABA_A_Rs containing the α5 subunits.

### α5-SOP002 Enhanced Memory in Healthy Rodents

**Figure 3:**
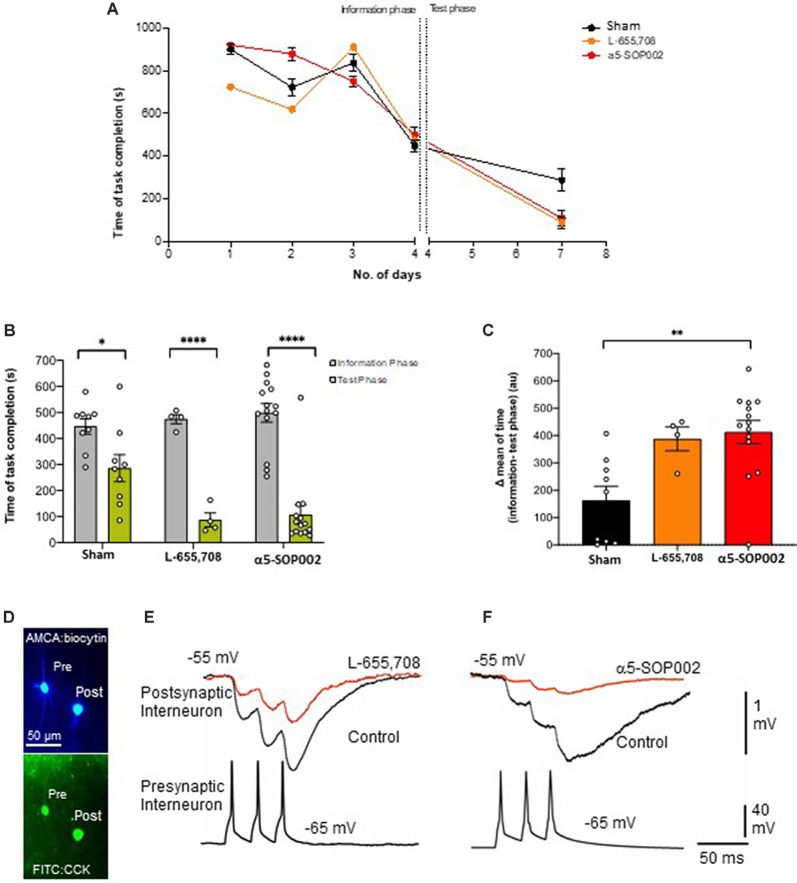
α5-SOP002 improved memory in healthy rats performing the eight-arm radial arm maze (RAM) test, consistent with the NAM effect shown by *in vitro* electrophysiological recordings. **(A–C)** Analysis of RAM test performed in healthy rats treated with “sham,” α5-SOP002- and L-655, 708. **(A)** Illustration of the variability of time taken to complete the memory task between all groups during 7 days (training + test phases). **(B)** Bar graphs compare the time taken to complete the tasks during training day 4 (defined as the information phase; gray) and day 7 (defined as test phase; green) for all groups tested. The time to complete the task during the test phase after administration of either; α5-SOP002 and L-655, 708, was reduced compared to control, suggesting potentiation of spatial memory recall. **(C)** Bar graphs representing the difference in mean time taken to complete the task between the information and test phase of each group. The a5-SOP002-treated rats had a statistically significant difference while both α5-SOP002- and L-655, 708-treated groups showed a bigger difference compared to the sham group. Results are expressed as mean ± standard error of the mean (SEM; black; sham group, *n* = 9, orange; L-6, 55 *n* = 4 and red; a5-SOP002 *n* = 14; one-way ANOVA, *P < 0.05, **P < 0.01, ****P < 0.0001). **(D)** Representative images of the anatomy of dendrite targeting, presynaptic CCK schaffer collateral-associated (SCA) cells synaptically connected to a postsynaptic CCK cell (AMCA shows the biocytin labeling during electrophysiological recordings). **(E,F)** Paired recording, trains of presynaptic action potentials elicited unitary inhibitory postsynaptic potentials (IPSPs), that were reduced by both NAMs selective for a5 GABA_A_ receptors, L-655, 708 and α5SOP002.

As a proof of concept, experiments were performed on healthy rats, to test the effects of α5-SOP002 *in vivo*, using the RAM memory test. Rats were divided into three groups according to the treatment they received, our compound α5-SOP002 (*n* = 14), the commercially available GABA_A_ α5 inverse agonist, L-655, 708 (*n* = 4; similar to α5IA), and saline-treated “sham” group (*n* = 9). During the first 3 days of the pre-treatment training phase, all groups took between 600 s and 800 s to complete the task and by the fourth day, the task was completed more efficiently within 450 s. The fourth day was considered as the information phase, assuming the rats have now learned the maze or gathered all the “information” to complete the task to a certain degree. All groups completed the task significantly faster on the test day in comparison to the information phase. The α5-SOP002- and L-655, 708-treated groups completed the task faster than the sham group on the test day taking almost three times less of the time and showed a bigger difference between information and test phase (Figures 3A–C). This validates our compound has an *in vivo* effect, potentially a memory-enhancing one.

### Preservation of α5 GABA_A_Rs in CA1 Pyramidal Cells and Three Sub-types of Interneurons in the AD Model

**Figure 4:**
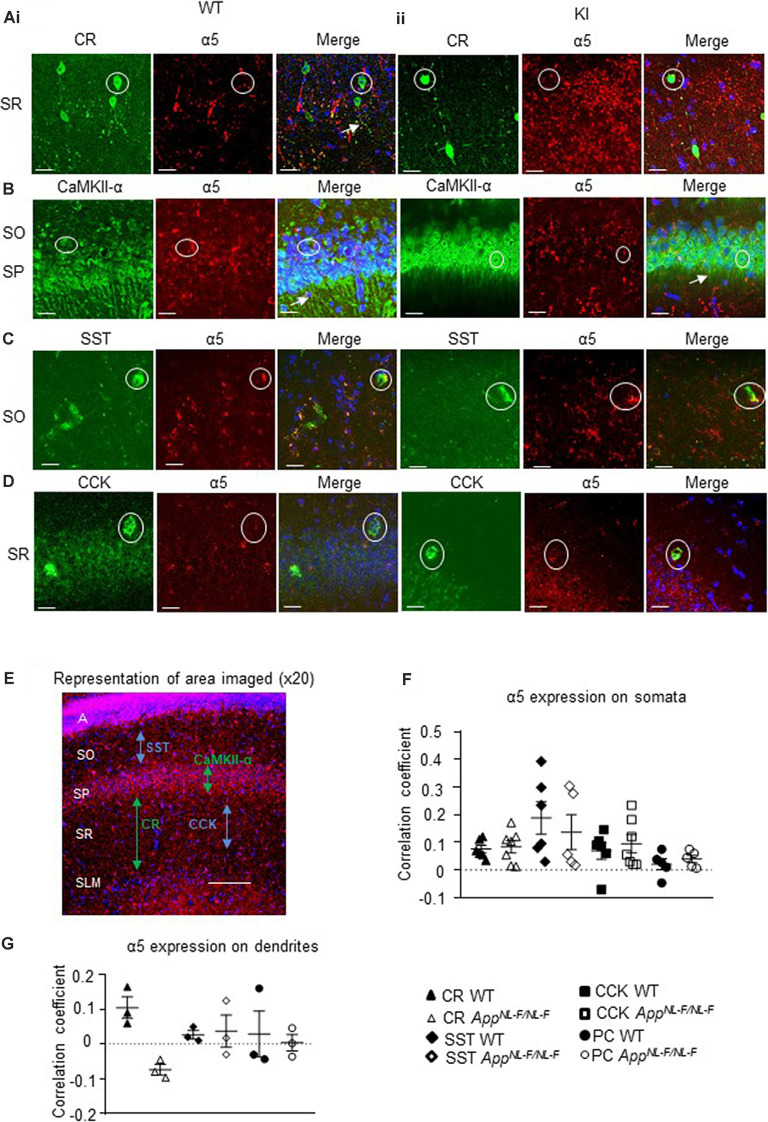
Expression of α5 subunit-containing GABA_A_Rs in CA1. **(A–D)** Confocal microscopy *Z*-stacks at 63× magnification showing α5 subunit-containing GABA_A_R expression (red, Alexa 488 or Alexa 568 label) on pyramidal neurons (CaMKII- α, green, FITC), CR interneurons (green, Alexa 488), SST interneurons (green, Texas Red), and CCK interneurons (green, Texas Red) in wild-type **(i)** and *APP*^NL−F/NL−F^ animals **(ii)**. Panels show individual channels and merged image with the nuclear stain DAPI (blue). Representative cell somata are outlined with white circles. White arrows indicate dendritic co-localization of α5. **(E)** Representative image labeled with α5 (red) and DAPI (blue) taken at 20× magnification in the CA1 of *APP*^NL−F/NL−F^ to exemplify the region of data acquisition, arrows indicate the location of the sub-types of cells imaged and analyzed. Layers are labeled: alveus **(A)**, stratum oriens (SO), stratum pyramidale (SP), stratum radiatum (SR), stratum lacunosum moleculare (SLM). **(F)** Analysis of α5 subunit-containing GABA_A_R expression on the soma of the four sub-types of neurons investigated. Each data point represents an average value (from five cells) analyzed form individual animals at 12–18 months of age (*n* = 5–7 mice studies per cohort). **(G)** Analysis of α5 subunit-containing GABA_A_R expression on the dendrites of CR cells, SST cells, and pyramidal neurons (*n* = 3 mice per genotype with visible proximal dendrites analyzed for five cells per animal). **(F,G)** Results are expressed as a scatter plot ± (SEM; results not significant, *P* > 0.05), of Pearson correlation coefficient as a measure of co-localization, after application of Fisher’s transformation. Data analyzed with a one-way ANOVA and *post hoc* Tukey test.

Using immunofluorescence and confocal microscopy analysis in the CA1 region of the hippocampus, we investigated α5 subunit-containing GABA_A_R expression in three sub-types of modulatory inhibitory interneurons, CR-, SST- and CCK-expressing interneurons, as well as in pyramidal cells (stained for CaMKII-α) in the *APP*^NL−F/NL−F^ mouse model and wild-type animals (Figures 4A–D). The imaged area in each case is shown in Figure 4E.

This was measures in three different ways, quantification of the total intensity of α5 signal in CA1 measured from confocal *Z*-stacks, followed by the quantification of α5 expression from individual cell populations measured from their somata and dendrites, using Pearson’s correlation coefficient R with Fisher’s transformation.

We also quantified the total intensity of α5 signal in CA1 confocal *Z*-stacks and observed no differences in the AD model compared to wild-type (*P* > 0.05, *n* = 5 wild-type animals and 6 *APP*^NL−F/NL−F^ animals), suggesting preservation of α5 expression in the *APP*^NL−F/NL−F^ animals (12–18 months age).

The α5 subunits expressed on all three interneuron subtypes were analyzed further from somata of the different cell types (Figure 4F). There was no significant change in α5 expression on CR cells in *APP*^NL−F/NL−F^ animals compared to wild-type (only a slight increase of 11.86 ± 3.14%, *P* > 0.05, *n* = 6 wild-type animals and 7 *APP*^NL−F/NL−F^ animals). Similarly, there was no change in the expression of α5 expression in SST or CCK interneurons between wild-type and *APP*^NL−F/NL−F^ mice (changes of; 27.35 ± 12.61% and 36.09 ± 12.45% observed in SST and CCK cells, respectively, in *APP*^NL−F/NL−F^ animals compared to wild-type animals, *P* > 0.05, *n* = 6). Thus, the three interneuron subtypes studies showed no significant differences in α5 subunit expression between wild-type animals and *APP*^NL−F/NL−F^ animals, highlighting the preservation of the α5 subunit in AD.

Analysis of CaMKII-α and α5 co-staining (Figure 4F) showed no significant differences in the expression of α5 expression on the pyramidal cells in *APP*^NL−F/NL−F^ animals compared to wild-type (*P* > 0.05, *n* = 5). This observation is consistent with previous studies, which reported α5 expression on pyramidal cells (Brünig et al., 2002).

Next, we investigated the expression of the α5 subunit on CR, SST, and pyramidal cell dendrites (Figure 4G), as the subunit has been reported to be located postsynaptically at dendritic sites where presynaptic CR cells target SST interneurons (Magnin et al., 2019) and on postsynaptic dendrites of pyramidal cells (Ali and Thomson, 2008). CCK cells also receive input-from dendrite-targeting interneurons (Ali, 2007), but their dendrites could not be investigated in detail here, due to the unavailability of a specific anti-CCK antibody that shows a good expression of CCK in dendrites in mouse tissue. We investigated up to 5 cells in each animal, and observed no significant difference in the α5 expression between the genotypes or neuron subtypes in their dendrites (*P* > 0.05, one-way ANOVA with *post hoc* Tukey’s test for multiple comparisons).

### α5-SOP002 “Normalizes” CR Interneuron Aberrant Inhibition Observed in AD

#### Inhibition Recorded From Spontaneous Synaptic Events

**Figure 5:**
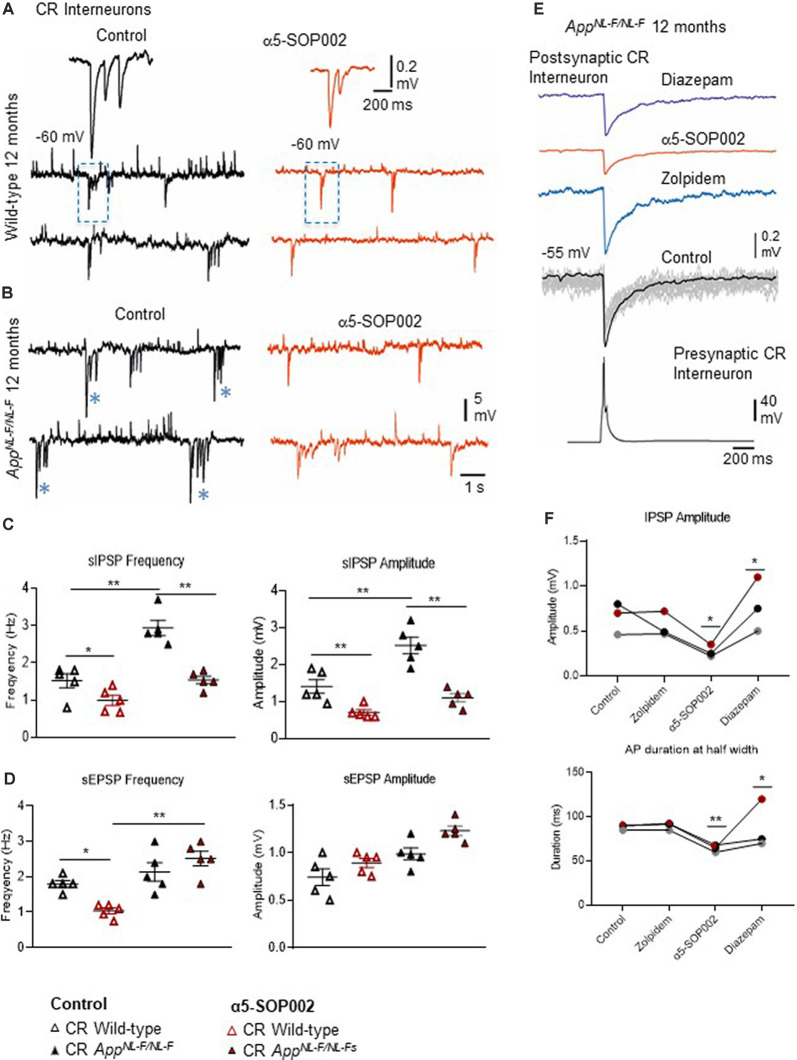
Calretinin (CR)-expressing interneurons are functionally restored by NAM of α5 subunit-containing GABA_A_Rs in *APP*^NL−F/NL−F^ mice. **(A,B)** Whole-cell current-clamp recordings of spontaneous inhibitory/excitatory postsynaptic potentials (sIPSPs and sEPSPs) recorded in CR cells in CA1 of 12-month-old wild-type and *APP*^NL−F/NL−F^ mice, at membrane potentials of −60 mV in control conditions, and after bath-application of α5-SOP002 (red traces). The squares indicate where synaptic events have been enlarged and shown in the inserts. *Indicate, an usually high sIPSPs recorded in the AD model. **(C,D)** Bar graphs show the average sIPSP and sEPSP amplitude and frequency at −60 mV in CR cells recorded in wild-type mice and the *APP*^NL−F/NL−F^ mouse model. These data suggest a significantly enhanced amplitude and frequency of inhibition in the AD model, which was “normalized” to control values after bath- application of α5-SOP002. **P < 0.01, Data analyzed with a two-way ANOVA and *post hoc* Tukey’s test. **(E)** Paired recording obtained between two putative CR cells recorded in SR of CA1 in the AD model. The unitary IPSPs were not sensitive to zolpidem, reduced by α5-SOP002, and then enhanced by subsequent addition of diazepam, indicating α5 pharmacology. **(F)** Line graphs show the average unitary IPSP amplitude and width at half amplitude change for each paired recording between two CR cells, in control, and after bath-application of zolpidem, α5-SOP002 and diazepam, recorded at −55 mV in *APP*^NL−F/NL−F^ mouse model. *P < 0.05, **P < 0.01. Data analyzed with a one-way ANOVA and *post hoc* Tukey test. Blue (*) are representative traces that have been enlarged in the inserts.

The effect of α5-SOP002 at inhibitory CR interneurons was determined on brain slices by performing whole-cell recordings under current-clamp mode. sIPSPs and sEPSPs were recorded from CR interneurons at 10–12 months old wild-type and *APP*^NL−F/NL−F^ mice at holding membrane potentials of −60 mV (to observe both excitation and inhibition; Figures 5A–D), the average data are shown in Table 1. The average peak frequency and amplitude of sIPSPs significantly increased in the AD model compared to wild-type age-matched mice at −60 mV, consistent with our previous publication that reported this interesting abnormal observation in the CR cells (Shi et al., 2019). In the *APP*^NL−F/NL−F^ mice, sIPSP frequency and amplitude were abnormally higher by 93.4 ± 7.5% (*P* < 0.01, *n* = 5, *n* = 5, two-way ANOVA with *post hoc* Turkey’s test) and 55.6 ± 23.3% (*P* < 0.01, *n* = 5) of control sIPSPs recorded in age-matched wild-type mice, respectively (Figure 5C).

**Table 1:** Changes of spontaneous synaptic events recorded in CR, CCK-SCA, and pyramidal cells after bath-application of a5-SOP002 in 10–12 months of age-matched, wild-type, and *APP*^NL−F/NL−F^ mice.

Cell subtype	CR cells *n* = 5		CCK cells *n* = 6		Pyramidal cells *n* = 5	
sIPSP frequency (Hz)	Control	α5-SOP002	Control	α5-SOP002	Control	α5-SOP002
Wild-type	1.52 ± 0.19	0.99 ± 0.14**	1.18 ± 0.02	0.81 ± 0.03**	1.14 ± 0.04	0.76 ± 0.06**
*APP*^NL−F/NL−F^	2.94 ± 0.20**	1.54 ± 0.10**	0.90 ± 0.03**	0.44 ± 0.05**	0.90 ± 0.02**	0.51 ± 0.02**
**sIPSP Amplitude (Hz)**						
Wild-type	1.41 ± 0.19	0.71 ± 0.07**	0.57 ± 0.02	0.26 ± 0.02**	1.01 ± 0.05	0.50 ± 0.02**
*APP*^NL−F/NL−F^	2.52 ± 0.23**	1.10 ± 0.11**	0.43 ± 0.02**	0.23 ± 0.02**	0.24 ± 0.03**	0.12 ± 0.02**
**sEPSP Frequency** **(mV)**						
Wild-type	1.8 ± 0.09	1.04 ± 0.09*	1.26 ± 0.03	1.96 ± 0.04**	1.52 ± 0.02	2.52 ± 0.09
*APP*^NL−F/NL−F^	2.14 ± 0.26	2.52 ± 0.20**	2.11 ± 0.5**	3.15 ± 0.06**	3.04 ± 0.06**	4.32 ± 0.05**
**sEPSP Amplitude (Hz)**						
Wild-type	0.74 ± 0.09	0.89 ± 0.05	0.68 ± 0.04	1.06 ± 0.04**	0.74 ± 0.02	1.34 ± 0.06**
*APP*^NL−F/NL−F^	0.98 ± 0.06	1.23 ± 0.05	0.89 ± 0.02**	2.00 ± 0.05**	2.00 ± 0.02**	3.44 ± 0.05**

**Figure 6:**
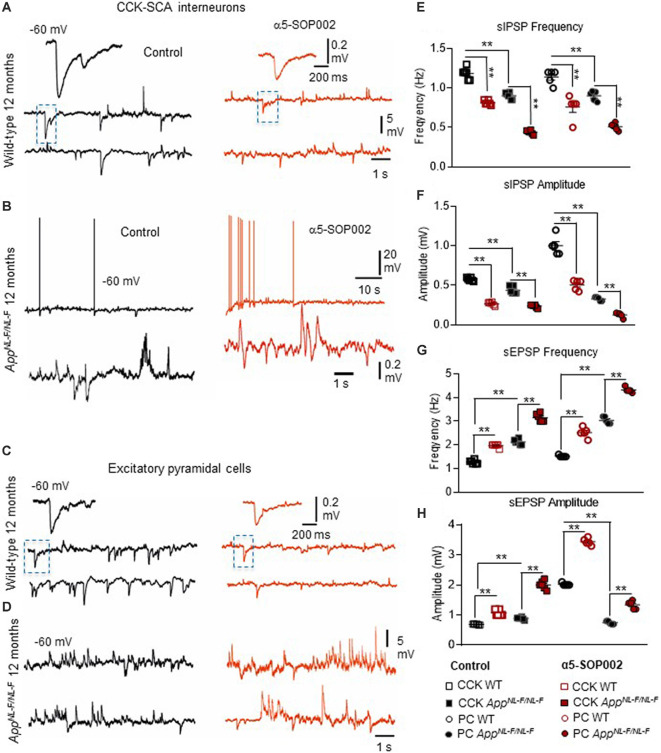
CCK interneurons and pyramidal cells are further compromised by NAM of α5 subunit-containing GABA_A_Rs in *APP*^NL−F/NL−F^ mice. **(A–D)** Whole-cell current-clamp recordings illustrating sIPSPs and sEPSPs recorded in CCK-SCA cells **(A,B)** and pyramidal cells **(C,D)** in CA1 of 12-month-old wild-type and *APP*^NL−F/NL−F^ mice, recorded at a membrane potential of −60 mV in control conditions and after bath-application of α5-SOP002. Bath-application of the α5-SOP002 resulted in a reduction in sIPSP amplitude and frequency, but also increased membrane excitation in both cell types, thus further increasing the aberrant hyperexcitability in the AD model. **(E–H)** Bar graphs show the overall pharmacological change after applying α5-SOP002 in CCK-SCA and pyramidal cells recorded from wild-type and *APP*^NL−F/NL−F^ mice at 10–12 months. **P < 0.01. Data analyzed with a two-way ANOVA and *post hoc* Tukey’s test, see Table 1 for details.

Averaged values in control and after bath-application of a5-SOP002 ± SEM are shown, significant difference indicated as with asterisk (*P < 0.05, **P < 0.01), indicate differences between data sets obtained before and drug application with the same genotype. Significant differences between genotypes are indicated with a blue asterisk (see also Figures 5, 6). A two-way ANOVA followed by *post hoc* Tukey’s test for multiple comparisons was used to determine the statistical value. The sample size n denotes the number of animals (one cell per animal was recorded in these experiments). sIPSP, spontaneous inhibitory postsynaptic potential. sEPSP, spontaneous excitatory postsynaptic potentials.

Bath-application of α5-SOP002 (1 μM) reduced the sIPSP frequency and amplitude in both wild-type and *APP*^NL−F/NL−F^ mice (see Table 1 for details). The significantly reduced sIPSP frequency (48 ± 3.2%, *P* < 0.01, *n* = 5, two-way ANOVA with *post hoc* Turkey’s test) and amplitude (56.3 ± 5.7%, *P* < 0.01, *n* = 5, two-way ANOVA with *post hoc* Turkey’s test) recorded in CR cells from *APP*^NL−F/NL−F^ mice was comparable to the control CR cells recorded in age-matched wild-type mice. The average sEPSP frequency and amplitude also changed, but the slight increase was not significantly different from the control mean (Figure 5D, Table 1).

Interestingly, in the *APP*^NL−F/NL−F^ mice, bath-application of α5-SOP002 also caused an average ~5 mV depolarization of the cell membrane, suggesting a reduction in tonic inhibition.

#### Unitary Inhibition Recorded From Two Synaptically-Connected CR Cells

CR interneurons during the late stages of AD were readily identifiable under ID-DIC during experiments (in striking contrast to CCK or SST cells that were not easily visualized), allowing us to perform paired recording between two CR cells. We performed paired recording in the *APP*^NL−F/NL−F^ animals only due to the very technically challenging nature of these experiments, hampered by the age of the mice. Figures 3E,F shows examples of paired recordings performed in younger healthy control rats where pre and postsynaptic cells were identified as CCK-positive (example of anatomy shown in Figure 3D).

Consistent with the finding that the sIPSPs recorded in “putative” CR cells (biocytin filled cells identified with a light microscope, and were not reconstructed), was sensitive to α5-SOP002, unitary IPSPs recorded between two CR cells in SR were also reduced in peak amplitude and width at half amplitude following bath-application of α5-SOP002 at −55 mV (Figure 5E). The decrease in amplitude and width was: 51.20 ± 7.36% (*P* < 0.05, *n* = 3, paired, two-tailed student’s *t*-test) and 28.25 ± 1.02% (*P* < 0.01, *n* = 3, paired, two-tailed student’s *t*-test) of control IPSPs recorded in *APP*^NL−F/NL−F^, respectively. Bath-application of the α1 subunit-selective agonist, zolpidem did not change the IPSP properties at these synapses, which was consistent with previous studies that reported insensitivity to zolpidem at synapses involving presynaptic dendrite-preferring cells (Ali and Thomson, 2008). Subsequent addition of the broad spectrum benzodiazepine site agonist, diazepam (after α5-SOP002) enhanced IPSP amplitude by 186.59 ± 41.45% (*P* < 0.05, *n* = 3, one-way ANOVA) and width at half amplitude by, 37.31 ± 6.71% (*P* > 0.05, *n* = 3, one-way ANOVA with *post hoc* Bonferroni’s test) of control IPSPs recorded in *APP*^NL−F/NL−F^ mice (Figures 5E,F).

The recorded (putative) CR-expressing interneurons, recovered *post hoc* were usually oval with two to three vertically orientated primary beaded dendrites, usually from opposite poles, with fine axons containing small/medium-sized boutons originated from the soma or a primary dendrite and ramified quite sparsely in mid-SR, as described previously (Shi et al., 2019) These cells resembled previously published CR cells (Gulyas et al., 1996).

### α5-SOP002 Reduced Inhibition but Exacerbated Synaptic Hyperexcitability at CCK and Principal Cells

We then attempted to record from CCK and pyramidal cells in CA1. The anatomically recovered interneurons resembled the most abundant subtype of CCK-expressing cells, the Schaffer collateral-associated (SCA) interneuron with soma/dendrites and axons predominantly located in the SR and axonal branches predominantly ramifying in SR (Ali, 2007). CCK and SST-expressing cells in aged AD mice decline in densities during the pathogenesis of AD (Shi et al., 2019), which hampered the yield of the recordings. Furthermore, we could not record from SST-expressing cells in SO due to their sparse appearance in the slices and the heavy myelination in this region at 10–12 months of age.

Bath application of the GABA_A_R α5 NAM, α5-SOP002, resulted in a general trend in reducing the average sIPSP amplitude and frequency recorded in both CCK-SCA and pyramidal cells in age-matched wild-type and *APP*^NL−F/NL−F^ mice (Figure 6, see Table 1 for detailed values), significant changes are indicated in Figure 6 and Table 1. In *APP*^NL−F/NL−F^ mice, the average sIPSP frequency and amplitude recorded at CCK-SCA cells reduced by 51.20 ± 1.00% and 46.18 ± 1.90%, of control values by bath- application of α5-SOP002 (*P* < 0.01, two-way ANOVA, with *post hoc* Tukey’s test, *n* = 5; Figures 6A,B,E,F). Similarly, in *APP*^NL−F/NL−F^ mice sIPSP frequency and amplitude recorded in pyramidal cells reduced following bath-application α5-SOP002, by 43.45 ± 1.76% (*P* > 0.05, *n* = 5, two-way ANOVA) and 62.19 ± 7.19% (*P* < 0.01, *n* = 5, two-way ANOVA with *post hoc* Tukey’s test) of control sIPSPs recorded in age-matched wild-type mice, respectively (Figures 6D,E,G,H).

However, in contrast, with bath-application of α5-SOP002, the sEPSP properties recorded in CCK-SCA and pyramidal cells significantly *increased* in both wild-type and *APP*^NL−F/NL−F^ mice (see Table 1). These cells recorded in the AD model displayed an abnormal level of hyperexcitation and a deficit in inhibition compared to the healthy, wild-type mice (Figures 6G,H; see also Petrache et al., 2019; Shi et al., 2019), which was further exacerbated when challenged with the GABA_A_R α5 NAM, α5-SOP002. With bath application of α5-SOP002, in the *APP*^NL−F/NL−F^ mice, the increase in sEPSP frequency and amplitude in CCK-SCA was 42.10 ± 0.47% and 70.29 ± 1.04% (*P* < 0.01, *n* = 6, two-way ANOVA with *post hoc* Tukey’s test), and in pyramidal cells was, 48.8 ± 0.94% and 124.71 ± 3.17% (*P* < 0.01, *n* = 6, two-way ANOVA with *post hoc* Tukey’s test), respectively.

## Discussion

In this study, we have focused on establishing whether the modulation of α5 GABA_A_R-associated synaptic transmissions by compounds with negative allosteric effects could be a successful targeted therapeutic strategy in AD.

It has been evidenced that the GABA_A_R α subunits form a structural basis for the different pharmacological and thus, behavioral profiles of various allosteric modulators of these receptors (Mohler et al., 2002; Whiting, 2003). In particular, allosteric modulation of α5-containing GABA_A_Rs has been shown to gate the acquisition and modify the extinction of associative learning in animal models (Collinson et al., 2002; Crestani et al., 2002; Yee et al., 2004; Dawson et al., 2006), while positive modulators of α5 GABA_A_R were also found to rescue hippocampal-dependent memory deficits in memory-impaired rats tested with water and radial-arm mazes (Koh et al., 2013). Yet clinical trials aimed at alleviating cognitive deficits with selective NAMs of these receptors have failed. Our objective in the current study was to resynthesize a hybrid compound of an established NAM, 6,6-dimethyl-3-(2-hydroxyethyl)thio-1-(thiazol-2-yl)-6,7-dihydro-2-benzothiophene-4(5H)-one, to increase its’ aqueous solubility, as well as its’ selectivity and potency as a NAM of α5 GABA_A_Rs. Inhibition mediated *via* these receptors is widespread in the brain but it is particularly abundant in the hippocampus (Magnin et al., 2019), where we have identified four sub-populations of neurons that express high levels of α5 GABA_A_Rs. Using the *APP*^NL−F/NL−F^ knock-in mouse model of AD, that shows an age-dependent increase in the main pathological hallmarks of this disease, including accumulation of Aβ, activation of microglia and reactive astrocytes and neurodegeneration (Shi et al., 2019), we have revealed how the negative allosteric modulation of α5 GABA_A_Rs can *exacerbate* the aberrant hyperexcitability and synaptic dysregulation in AD.

### Mechanism of Action of Our Key Compound α5-SOP002

From computational modeling, we showed that α5-SOP002 docked into the interface of the α5 and γ2 subunits, indicating that it works *via* the benzodiazepine binding site (composed of a γ2 and either α1, α2, α3, or an α5 subunit of the GABA_A_R). Normally, binding of benzodiazepines to these sites causes a conformational change of the receptor increasing the receptor’s affinity for GABA, resulting in an enhanced inhibitory (hyperpolarizing) effect mediated *via* Cl^−^ flux (Sieghart, 1995). However, NAMs, such as α5-SOP002, when bound to the same GABA_A_R sub-types *decrease* the influx of Cl^−^ which leads to depolarization of the membrane and a *decreased* net inhibitory effect (Haefely et al., 1993). The data obtained from various HEK cell-lines constructed to contain specific GABA_A_R subunits and electrophysiological recordings performed, provided evidence to suggest that the developed compound, α5-SOP0002 specifically acted as a NAM at α5 GABA_A_Rs and had no effect on α1 or α2 subunit-containing GABA_A_Rs. However, this does not preclude an action of α5-SOP0002 as a NAM in native GABA_A_Rs where the synaptic colocalization of the α subunits could result from a combination of the insertion of either two identical α subunits, or from insertion of a single receptor sub-type that contains two differentα subunits. Theα subunit that is adjacent to the γ2 subunit dominates the pharmacological profile of the receptor as suggested previously by binding studies on double immunopurified α1/α5 GABA_A_Rs (Araujo et al., 1999). Thus, we suggest that α5-SOP002 acts by specifically binding at the interface of α5 and γ2 subunits, which determines a unique pharmacological profile of this compound.

### Preservation of α5 GABA_A_Rs in CA1 in the Aged Mouse Model of AD

We show for the first time, that the α5 GABA_A_Rs in the CA1 region of the hippocampus are expressed on CR-expressing interneurons, specialized for dis-inhibition, but also SST- and CCK-expressing interneurons, specialized for fine-tuning pyramidal cell activity. The rationale for selecting CCK- and SST-expressing cells in our experiments stems from previous studies showing that dendrite-targeting interneurons form synapses with the pyramidal cells that incorporate the α5 subunit-containing GABA_A_Rs (Ali and Thomson, 2008). However, in the current study, we show that SST- and CCK-expressing cells are also recipients of postsynaptic inhibition mediated by α5 GABA_A_Rs.

Our findings corroborate previous studies that have demonstrated that α5 GABA_A_Rs are preserved in post-mortem tissue obtained from AD patients (Howell et al., 2000; Palpagama et al., 2019), but also studies showing expression of α5 GABA_A_Rs in pyramidal cells (Brünig et al., 2002). Our experiments demonstrate the expression of these receptors on the soma of CR, SST, and CCK interneurons in addition to pyramidal cells. However, the expression pattern of α5 GABA_A_Rs in our study was in contrast to previous studies that show more diffuse staining in SR and SO compared to the pyramidal cell layer, which showed less expression of these receptors (Houser and Esclapez, 2003; Atack et al., 2005; Vidal et al., 2018). These differences could be attributed to the specificity of the antibodies, experimental protocol, or the disease model under investigation.

Since SST and CCK cells decline in the *APP*^NL−F/NL−F^ knock-in mouse model of AD (Shi et al., 2019), this distribution could be due to a subgroup of SST interneurons compensating for the reduction in numbers by upregulating α5 GABA_A_R expression, interestingly, some studies show upregulated α5 subunits in SP and oriens of the CA1 region (Kwakowsky et al., 2018). Given that both CCK and SST cells are hyperactive in AD (Zhang et al., 2016; Shi et al., 2019), possibly the α5 expression represents a compensatory mechanism. An investigation into the levels of α5 expression on dendrites showed larger variability, notable being the level of expression on SST interneurons in the *APP*^NL−F/NL−F^ mice, which could be linked to the differential input those cells receive. Similarly, pyramidal cells showed larger variability, and we propose that this is input-dependent. Earlier studies investigating the regulation of GABA_A_R surface expression show that, during seizures, receptors can be rapidly internalized leading to increased neuronal activity (Goodkin et al., 2007). A similar mechanism could be taking place in AD, contributing to the abnormal inhibitory-excitatory balance that characterizes this disease (Petrache et al., 2019).

### Aberrant Inhibition in CR Interneurons Staining of in This Study Differs From Is “Normalized” by α5-SOP002 in the AD Model

Previously, we reported that the CR interneuron network was “preserved” in our AD model following post-phenotypic changes such as increased Aβ accumulation and proliferation of microglial cells and astrocytes, which is consistent with anatomical studies reporting resilience of CR cells in post-mortem brains of AD patients (Fonseca and Soriano, 1995). Furthermore, the inhibitory parameters elicited in CR cells recorded in the AD models were abnormally enhanced compared to control mice, which was consistent with our previous study that suggests the involvement of the P2Y1 purinoreceptor system in regulating the abnormal inhibition expressed amongst the CR interneuron network (Shi et al., 2019). In this study, using our key NAM molecule, α5-SOP002, we have demonstrated that abnormal synaptic inhibition received by CR interneurons in the *APP*^NL−F/NL−F^ mouse model “normalized” to control levels. Moreover, paired whole-cell recordings revealed that α5-SOP002 had a pronounced effect at synapses between interneurons compared to synapses received by pyramidal cells, therefore impacting on dis-inhibition in the hippocampal CA1 region. This is important, given that we have previously demonstrated a gradual decline in the number of CCK- and SST-inhibitory interneurons in our AD model, suggesting an overall reduction in their inhibitory function, which was in stark contrast to the density of CR cells (Shi et al., 2019).

The sIPSPs recorded in this study are most likely due to the activation of synaptic α5 GABA_A_Rs since we did not observe any significant change in either membrane potential or input resistance associated with the application of α5-SOP002 onto CR interneurons (or neither CCK nor pyramidal cells). We suggest that in the CR interneuron network, showing zolpidem insensitivity, augmentation by diazepam, and depression by α5–SOP002, the α5 subunit may coexist with another α5 subunit or either α2 or α3- subunit, where α5 pharmacology predominates.

However, interestingly, we observed a small positive (depolarization) change in membrane potential in CR interneurons with α5-SOP002 in the AD model only, suggesting that these cells may be in a state of excess tonic inhibition in the disease state. We suggest that the release from the abnormal tonic inhibition at CR cells, indicated by the depolarization of the membrane potential, could be caused by negative allosteric modulation of extrasynaptic α5-receptors (Caraiscos et al., 2004; Magnin et al., 2019), which are tonically active due to increased levels of ambient GABA (Scimemi et al., 2005). Given that α5-SOP002 requires the presence of α- and γ-subunits, it is unlikely that it can affect the activity of other types of extrasynaptic GABA_A_Rs such as those containing the δ-subunit. However, the contribution of extrasynaptic α5 GABA_A_Rs to the CR interneuron network remains to be fully investigated.

#### Negative Allosteric Modulation of α5 Subunit-Containing GABA_A_Rs Further Exacerbates Hyperexcited Synapses in the AD Model

As previously described, there is a gradual decline in the number of CCK-SCA interneurons and CaMKII-expressing pyramidal cells in aged AD mice, with the later showing hyperexcitability when the pathological hallmarks of AD were present, clearly indicating the abnormalities in neuronal network activity (Shi et al., 2019). Since these cells express the α5 subunit, it is not surprising that α5-SOP002 can reduce inhibition at CCK and pyramidal cells, and therefore exacerbate imbalance between the excitation and inhibition at these key neuronal populations in CA1 and impact on the efficacy and precision of the fine-tuning inhibition at both temporal and spatial domains. These are reasonable assumptions, since; CCK-SCA cells, which are ideally positioned to modulate CA3 input (Iball and Ali, 2011), and are important for fine-tuning individual neurons by retrograde cannabinoid signaling (Katona et al., 1999; Ali, 2007), whereas the SST, that fine-tune distal inputs received by CA1 pyramidal cells (Leao et al., 2012; Magnin et al., 2019), and are important for coordinating neuronal assemblies and gating of memory formation (Tort et al., 2007; Cutsuridis and Wennekers, 2009). Due to the prime location of these interneurons, it is feasible to suggest that both of these interneuron subpopulations may be involved in routing information flow to CA1 from CA3 and entorhinal cortex- pathways that are important for memory acquisition and retrieval, and their destruction during the pathogenesis of AD may be a significant contributing factor to cognitive decline. This is further supported by recent studies that show SST interneuron dysfunction triggered by amyloid β oligomers underlies hippocampal oscillation important for memory functions (Chung et al., 2020).

## Conclusion

In summary, using a multi-disciplinary approach, we have developed a novel, selective NAM for α5 GABA_A_Rs and characterized its effects on hippocampal dis-inhibition in a well-established mouse model of AD. We have shown that this modulator can “normalize” abnormal, inhibitory synaptic activity received by CR interneurons in this model, suggesting initially its’ therapeutic potential. Furthermore, our data provide evidence that α5 GABA_A_Rs are also preserved in other types of interneurons, such as CCK, SST, and CR interneurons.

Since our data suggest that α5 GABA_A_Rs are widely expressed by both dysfunctional and resilient neurons, and also that α5-SOP002 can compromise further the aberrant hyperexcitable network in the AD model, we propose that pharmacological modulation of α5 subunit-containing GABA_A_R networks may not be a suitable therapeutic target for cognitive impairment in AD. Although the evidence suggests an overall improvement of memory with GABA_A_ α5 inverse agonists in rodents, it is yet to be established what kind of short- and long-term effects these compounds might have in patients. We propose that the lack of specificity and efficacy in clinical trials could be at least in part due to a wide expression of α5 GABA_A_Rs in the hippocampus, both by various types of interneurons and pyramidal cells. Thus, targeting the α5 subunit with NAMs would result in a global effect on the hippocampal networks and would lack the specificity required to restore the complex network alteration during the pathogenesis of AD that leads to the observed excitatory-inhibitory imbalance.

## Data Availability Statement

All datasets presented in this study are included in the article/Supplementary Material.

## Ethics Statement

The animal study was reviewed and approved by British Home Office and UCL ethics committees.

## Author Contributions

AP: performed immunofluorescence studies on mouse brains to characterize the expression of α5 GABA_A_Rs in different subtypes of interneurons and assisted in preparing the manuscript. AK: designed and produced the new α5 cell line and assisted in preparing the manuscript. AM: synthesized and refined various analogs of α5 NAMs with varying biological activity. MN: designed and produced the α5β2γ2-GABA_A_R stable cell line. MK-S: performed molecular docking and identification of the final conformation of the developed NAM. SHa: computational modeling and assisted in preparing the manuscript. SHi: developed, refined, and synthesized the α5 compounds. JJ: supervised production and characterization of all HEK293 cell lines stably expressing GABA_A_Rs which were used in this study. AA: designed and coordinated the project, performed and analyzed all electrophysiology and neuroanatomy experiments, and prepared the manuscript. All authors contributed to the article and approved the submitted version.

## Conflict of Interest

The authors declare that the research was conducted in the absence of any commercial or financial relationships that could be construed as a potential conflict of interest.

## Footnotes

^1^http://www.rcsb.org./pdb

^2^www.molsoft.com

## Abbreviations

ACSF: Artificial cerebrospinal fluid
AD: Alzheimer’s disease
CCK: Cholecystokinin
CR: Calretinin
DAB: 3–3-diaminobenzidine
DMSO: Dimethyl sulfoxide
GABA: γ-Aminobutyric acid
IPSP: Inhibitory postsynaptic potential
PB: Phosphate buffer
PBS: Phosphate-buffered saline
PFA: Paraformaldehyde
NAM: Negative allosteric modulator
RT: 10–90% rise time
SCA: Schaffer collateral-associated
SR: Stratum Radiatum
sEPSP: Spontaneous excitatory postsynaptic potential
sIPSP: Spontaneous inhibitory postsynaptic potential
SEM: Standard error of the mean
TBS-T: Triton X-100 in Tris-buffered saline.
