# Supplementary material for: Selective Modulation of α5 GABAA Receptors Exacerbates Aberrant Inhibition at Key Hippocampal Neuronal Circuits in APP Mouse Model of Alzheimer’s Disease
Source: Front Cell Neurosci. 2020 Nov 11;14:568194. doi: 10.3389/fncel.2020.568194 (PMC7686552; doi:10.3389/fncel.2020.568194)
Supplement: Supplementary file 1 [file Data_Sheet_1.docx]

**Supplementary Files**

**Supplementary Methods 1**

**Scheme 1 – Chemical Synthesis of α5SOP002**

**5-Methylisoxazole-3-carbohydrazide (2)^1^**

Hydrazine (6.07 mL, 106 mmol) was added to a solution of methyl 5-methylisoxazole-3-carboxylate (**1**) (3 g, 21.26 mmol) in MeOH (37 mL) over 5 minutes at 0 °C. The resulting solution was stirred at 0 °C for a further 10 minutes and then for 1 hour at room temperature. The mixture was then re-cooled to 0 °C for 1 hour and the colourless crystals that formed were filtered to give 5-methylisoxazole-3-carbohydrazide (**2**) as a colourless solid (1.28 g, 9.07 mmol, 43%); 1H-NMR (500 MHz, CDCl3): δ 8.13 (bs, 1 H), 6.44 (s, 1 H), 4.08 (bs, 2 H), 2.48 (s, 3 H); 13C-NMR (125 MHz, CDCl3): δ 171.35, 160.19, 157.53, 101.50, 12.45.

**3-(6-Chloro-[1,2,4]triazolo[3,4-a]phthalazin-3-yl)-5-methylisoxazole (4)^2^**

A solution of 5-methylisoxazole-3-carbohydrazide (**2**) (1 g, 7.09 mmol) and 1,4-dichlorophthalazine (**3**) (1.41 g, 7.09 mmol) in dioxane (25 mL) was heated at reflux overnight. The solution was concentrated to about 2/3 of the volume under reduced pressure and the solid that formed was filtered and washed with a solution of dichloromethane/petroleum ether (1/3) to give 3-(6-chloro-[1,2,4]triazolo[3,4-a]phthalazin-3-yl)-5-methylisoxazole (**4**) as a colourless solid (1.87 g, 6.55 mmol, 92%); 1H-NMR (500 MHz, CDCl3): δ 8.92 (d, 1 H, J= 8 Hz), 8.36 (d, 1 H, J= 8 Hz), 8.11 (t, 1 H, J= 7.75 Hz), 7.99 (t, 1 H, J=7.75 Hz), 6.90 (s, 1 H), 3.70 (s, 1 H), 3.49 (s, 1 H), 2.60 (s, 3 H); 13C-NMR (125 MHz, CDCl3): δ 170.82, 152.04, 151.49, 135.58, 132.58, 127.87, 124.69, 123.17, 122.76, 101.94, 67.25, 12.50.

**5-Methyl-3-(6-(pyrazin-2-ylmethoxy)-[1,2,4]triazolo[3,4-a]phthalazin-3-yl)isoxazole (6)**

Pyrazin-2-ylmethanol (**5**) (50.1 mg, 0.455 mmol) was dissolved in anhydrous DMF (1.0 mL) at 0 °C and lithium bis(trimethylsilyl)amide (0.42 mL, 0.420 mmol) was added slowly. 3-(6-Chloro-[1,2,4]triazolo[3,4-a]phthalazin-3-yl)-5-methylisoxazole (**4**) (100 mg, 0.350 mmol) was added in one portion to the solution and the mixture stirred overnight at room temperature. The reaction mixture was diluted with water and the solid that formed was collected by filtration and washed with water (3 x 5 mL) and dried to give crude 5-methyl-3-(6-(pyrazin-2-ylmethoxy)-[1,2,4]triazolo[3,4-a]phthalazin-3-yl)isoxazole (**6**) (96 mg, 0.267 mmol, 76%); ^1^H NMR (400MHz ,CDCl3) δH ppm = 9.07 (1 H, d, J = 1.5 Hz), 8.67 - 8.59 (1 H, m), 8.59 - 8.48 (2 H, m), 8.28 - 8.15 (1H, m), 7.96 - 7.84 (1 H, m), 7.82 - 7.71 (1 H, m), 6.75 (1 H, d, J = 0.8 Hz), 5.75 (2 H, s), 2.52 (3 H, d, J = 1.0 Hz); ^13^C NMR (101MHz ,CDCl3) δC ppm = 170.0, 145.2, 144.6, 144.3, 141.8, 134.2, 131.1, 125.1, 124.3, 123.6, 101.6, 68.6, 12.3.

**5-Methyl-3-(6-(pyrazin-2-ylmethoxy)-[1,2,4]triazolo[3,4-a]phthalazin-3-yl)isoxazole hydrochloride (7)**

5-Methyl-3-(6-(pyrazin-2-ylmethoxy)-[1,2,4]triazolo[3,4-a]phthalazin-3-yl)isoxazole (**6**) (45 mg, 0.125 mmol) was taken up in DCM (10 mL) and Na2SO4 added. The solution was filtered and the solid washed with DCM (3 x 2 mL). The filtrate was treated with HCl (2 mL of a 1.0 M solution in diethyl ether, 2.00 mmol), whereupon a white precipitate formed. The mixture was diluted with Et2O (10 mL) and the solid collected by filtration and washed with Et2O (3 x 5 mL) and dried to give 5-methyl-3-(6-(pyrazin-2-ylmethoxy)-[1,2,4]triazolo[3,4-a]phthalazin-3-yl)isoxazole hydrochloride (**7**) as a colourless solid (42 mg, 0.106 mmol, 85%); ^1^H NMR (400MHz ,CDCl3) δH ppm = 9.07 (1 H, d, J = 1.5 Hz), 8.67 - 8.59 (1 H, m), 8.59 - 8.48 (2 H, m), 8.28 - 8.15 (1H, m), 7.96 - 7.84 (1 H, m), 7.82 - 7.71 (1 H, m), 6.75 (1 H, d, J = 0.8 Hz), 5.75 (2 H, s), 2.52 (3 H, d, J = 1.0 Hz); ^13^C NMR (101MHz ,CDCl3) δC ppm = 170.0, 145.2, 144.6, 144.3, 141.8, 134.2, 131.1, 125.1, 124.3, 123.6, 101.6, 68.6, 12.3.
